# Peripheral Giant Cell Granuloma Associated with a Dental Implant: A Case Report and Review of the Literature

**DOI:** 10.1155/2015/697673

**Published:** 2015-03-16

**Authors:** Amy Louise Brown, Paulo Camargo de Moraes, Marcelo Sperandio, Andresa Borges Soares, Vera Cavalcanti Araújo, Fabrício Passador-Santos

**Affiliations:** ^1^Department of Pathology, São Leopoldo Mandic Institute and Research Centre, 13045-755 Campinas, SP, Brazil; ^2^Department of Oral Medicine and Oral Surgery, São Leopoldo Mandic Institute and Research Centre, 13045-755 Campinas, SP, Brazil

## Abstract

The peripheral giant cell granuloma (PGCG) is a nonneoplastic lesion commonly caused by local irritation. This report describes a 46-year-old Caucasian male who presented with a PGCG associated with a dental implant. The dental implant was originally placed in August 2012. Ten months later, the patient presented with a well-circumscribed lesion associated with and covering the implant, at which time the lesion was excised. Four months later, due to recurrence of the lesion, a deeper and wider excisional biopsy with curettage of the adjacent bone was performed. No evidence of recurrence has been reported after 12 months of follow-up. Immunohistochemistry, using the antibody CD68, was performed to investigate the origin of the multinucleated giant cells, with their immunophenotype being similar to those of other giant cell lesions, including central giant cell granuloma, foreign-body reactions, and granulomatous reactions to infectious agents.

## 1. Introduction

The peripheral giant cell granuloma (PGCG) is a nonneoplastic lesion, characterized by reactive hyperplasia in the presence of local irritation [1, 2], which exhibits microscopic features that closely resemble the central giant cell granuloma (CGCG). In most cases, no causal factor is identified; however, dental calculus, followed by ill-fitting dentures and tooth fracture are the most common [3]. The clinical differential diagnosis of a reactive lesion of the gingiva must include pyogenic granuloma, traumatic fibroma, and peripheral ossifying fibroma.

There is little data in the literature regarding the prevalence of reactive lesions associated with dental implants and whether they develop due to mechanical or biological irritation, with the former being associated with inappropriate implant placement and the latter with poor oral hygiene. The complications that may arise in and around implant sites are numerous and may include dehiscence, mucositis, gingival hyperplasia, and the formation of a biofilm [4–6].

This case report describes a peripheral giant cell granuloma associated with a dental implant and reviews similar cases published in the English literature.

## 2. Case Presentation

**Figure 1:**
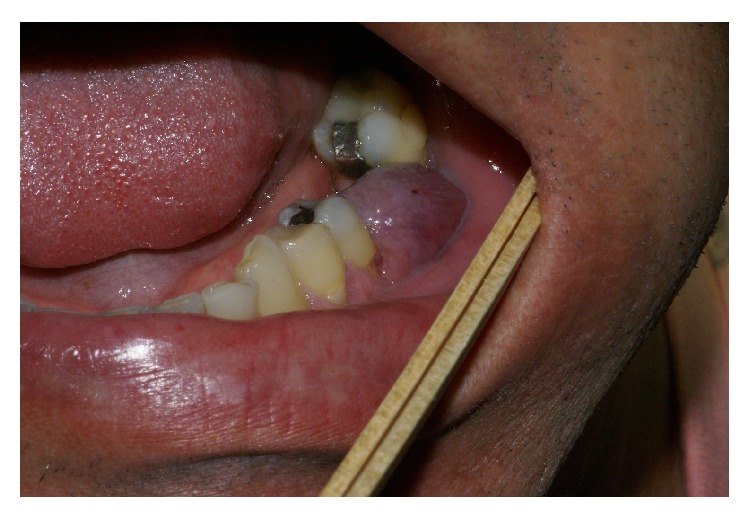
Clinical photograph of the painless purple pedunculated lesion associated with the dental implant.

**Figure 2:**
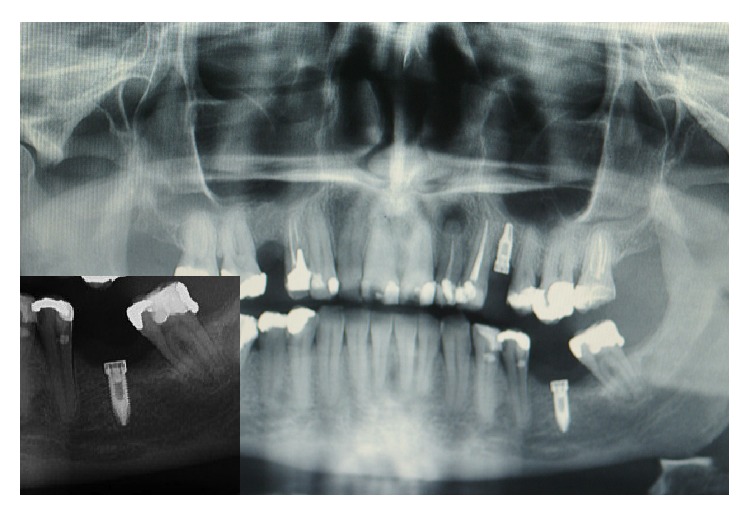
Panoramic radiograph showing the presence of a dental implant in the lower left molar region. Insert: increased magnification focusing on the implant in the region of the lower left molar, showing lack of radiographic features that would be compatible with bone involvement.

**Figure 3:**
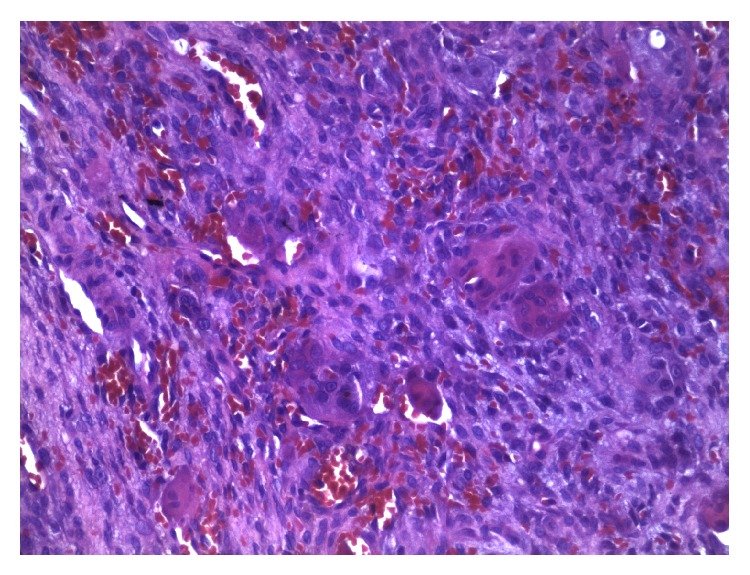
Photomicrograph revealing fragments of dense connective tissue, showing proliferation of ovoid and spindle-shaped cells, multinucleated giant cells, and congested blood vessels (haematoxylin and eosin stain, original magnification ×200).

A 46-year-old Caucasian male presented to the Department of Implant Dentistry, Faculty of Dentistry, University of São Leopoldo Mandic, Campinas, São Paulo, Brazil, in August 2012, requesting implants for aesthetic and functional purposes. Titamax Ex external hexagon implants (Neodent, Brazil) were placed in the regions of the upper left premolar and the lower left first molar, with their respective healing abutments. In June 2013, the patient presented with a lesion associated with and covering the lower left first molar implant site. Intraoral examination showed a well-circumscribed, pedunculated, painless, purple mass measuring approximately 1 cm, rubbery in consistency. Radiographically, in the lower left molar region, the presence of an implant was observed without evidence of radiographic features that would be compatible with bone involvement (Figure 2). The clinical differential diagnosis was pyogenic granuloma and peripheral giant cell granuloma (PGCG) (Figure 1). The patient underwent an excisional biopsy, which was sent to the Department of Oral Pathology, Faculty of Dentistry, University of São Leopoldo Mandic, Campinas, São Paulo, Brazil. The biopsy was fixed in 10% buffered formalin for 24 hours. Macroscopic examination revealed two fragments of brown-colored soft tissue, with a fibrous consistency, the larger fragment measuring 10 × 8 × 4 mm and the smaller fragment 7 × 6 × 3 mm. Histopathological examination revealed a fragment of mucosa lined by a parakeratinized stratified squamous epithelium. The lamina propria was composed of connective tissue containing various multinucleated giant cells surrounded by ovoid and spindle-shaped mesenchymal cells with multiple interspersed small blood vessels (Figure 3). The histopathological diagnosis was peripheral giant cell granuloma.

In October 2013, the patient was referred to the Oral Medicine Clinic, Faculty of Dentistry, University of São Leopoldo Mandic, Campinas, São Paulo, Brazil, with a recurrence of the lesion. A deeper and wider excisional biopsy, curettage of the adjacent bone, and application of surgical cement were performed. The biopsy was once again forwarded to the Department of Oral Pathology, Faculty of Dentistry, University of São Leopoldo Mandic, Campinas, São Paulo, Brazil. The histopathological diagnosis was peripheral giant cell granuloma. The patient remains lesion-free following one year of follow-up.

The paraffin-embedded blocks from each biopsy were selected for immunohistochemical staining using the antibody CD68. Five *μ*m sections were deparaffinized, hydrated, and subsequently immersed in 3% hydrogen peroxide for 30 minutes (Dinâmica, Diadema, SP, Brazil). For antigen retrieval, the slides were put into a steamer immersed in a citrate buffer (pH 6.0) for one hour at 95°C (Sigma, St. Louis, MO, USA). The sections were then incubated with the primary antibody overnight at 4°C at a dilution of 1 : 1200 (Dako, Carpinteria, CA, USA). The sections were then incubated with Labeled Streptavidin Biotin (LSAB, Dako, Carpinteria, CA, USA) for 30 minutes, stained for 5 minutes at 37°C with 3.3′-diaminobenzidine tetrachloride (Dako, Carpinteria, CA, USA), and counterstained with haematoxylin (Dinâmica, Diadema, SP, Brazil). Macrophages were used as the positive control.

**Figure 4:**
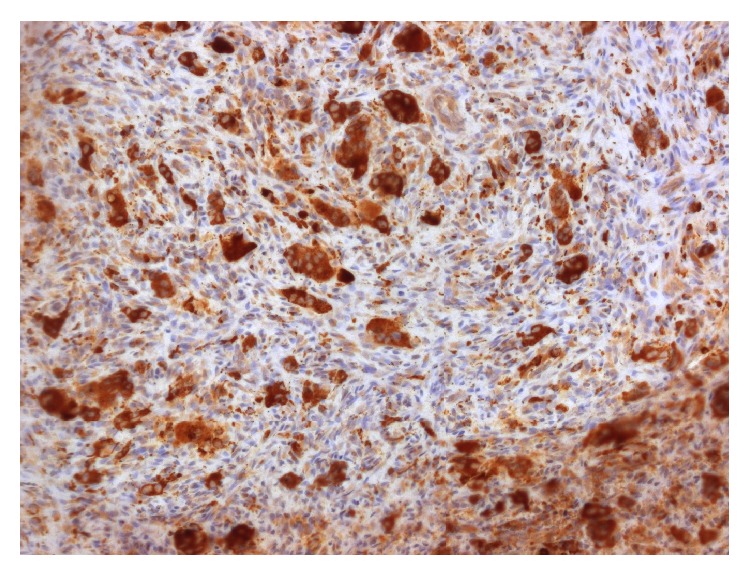
Immunohistochemical staining with CD68 showing strong positivity for multinucleated giant cells (original magnification ×200).

Immunohistochemical staining with CD68 was strongly positive for multinucleated giant cells, as shown in Figure 4, suggesting a histiocyte/macrophage or osteocyte origin.

## 3. Discussion

When compared with peri-implantitis, reactive lesions associated with dental implants, such as PGCG, are considered rare, with only 12 cases reported in the English literature (Table 1) [5, 7–13].

Dental implants aside, pyogenic granuloma is considered more common than PGCG; it is, therefore, extremely interesting that PGCG is in fact more common than pyogenic granuloma in the dental implant population. A search of the English literature (available for download) revealed 12 cases of PGCG and only five cases of pyogenic granuloma associated with dental implants [5, 6, 14–16]. Hirshberg et al. [7] revealed the presence of PGCG in 12% of 25 peri-implant soft biopsies examined.

Although the case presented in this study refers to a male patient, our review of the literature revealed that, as PGCG is not associated with dental implants, there is a female preference [1–3, 17], with implant-associated PGCG at a ratio of 1 : 1.6 (male : female). Günhan et al. [18] suggested that this may be due to the influence of sex hormones, with multinucleated giant cells being a target for oestrogen. Immunohistochemical investigation for oestrogen receptors in peripheral and central giant cell lesions of the jaws revealed positivity for the former and negativity for the latter, respectively [19, 20]. However, it may be suggested that in the context of implant-associated PGCG the influence of oestrogen would be less important, owing to the majority of patients being postmenopausal and hence serum levels of oestrogen fall.

In the present review, the age range was noted to be 31–74 years, with an average age of 50.9 years, as observed by Katsikeris et al. [21] who described PGCG as most common in patients aged 40 to 60 years. Motamedi et al. and Shadman et al., however, observed a mean age range of 31 and 33 years, respectively [2, 3]. It is important to highlight that the expected age range and average age may be higher in the implant population.

PGCG has been reported as being more common in the mandible [21], which can be corroborated by the present study, where the posterior mandible was demonstrated as the most common site, featuring eight of the 13 (62%) reported cases. The time lapse between implant placement and initial presentation of PGCG ranged from 3 months to 12 years, with the present case presenting 10 months after implantation. Although, controversial, tooth extraction has been described as an etiological factor for the development of PGCG [21, 22], as demonstrated by Hirshberg et al. [7], who showed that PGCG developed following tooth extraction in 8 to 11% of PGCG cases examined, appearing up to one year after the procedure. Therefore, it would be interesting to investigate whether the cases shown in Table 1 underwent tooth extraction prior to implant placement and, if so, the time between extraction and implant placement. A review of the literature revealed that dental implant-associated PGCG initially presented as peri-implantitis, loose healing abutments, exophytic masses, and profuse bleeding on toothbrushing. Cloutier et al. suggested that with time the abrasive surface of a dental implant has the capacity to become a source of chronic irritation, which, in some cases, may provoke reactive lesions, such as PGCG and PG [9].

In the cases reviewed, a recurrence rate of 46.2% was observed, with some lesions reported as recurring five times [11], which is considerably higher than that of PGCG not associated with dental implants, where a recurrence rate of 9.9% has been reported [22]. However, it is important to highlight that the number of cases reported so far is too small for valid conclusions to be made.

Six of 13 cases recurred, three of which had initially been treated solely with curettage. Only one case was excised from the start, which also recurred. Of the seven cases that were initially excised and curetted, only two recurred. Five of the thirteen cases required surgical removal of the implant (38%).

Bischof et al. [8] described a PGCG in a 56-year-old female with three implants in the posterior mandible. Despite correct spacing, the angulation of the implants was inappropriate, which led to the healing abutments of two of them to be partially unscrewed and juxtaposed. In addition, the patient described difficulty with performing oral hygiene, due to bleeding; therefore, dental plaque was allowed to accumulate, potentiating the tissue reaction. This corroborates the study by Özden et al. [10], who described a case of PGCG associated with a dental implant that occurred due to a poorly adapted prosthesis, which led to the accumulation of dental plaque and irritation of the gingiva. In the present study, a Titamax Ex external hexagon implant (Neodent, Brazil) was used, which is the material most commonly encountered in dental implants. Wilson Jr. et al. [23] demonstrated, using scanning electron microscopy (SEM) and energy dispersive X-ray spectrometer (EDS), that radiopaque foreign bodies, predominantly titanium and dental cement, surrounded by a chronic inflammatory infiltrate were present in 34 of 36 cases of peri-implantitis biopsied. However, in the present case, where only light microscopy was used, no particles were observed.

The presence of peri-implant metal particles has been reported as being caused by the insertion mechanics, an inadequate abutment placement, and early removal of failing implants [24], all of which may cause a hypersensitivity reaction and subsequent macrophage recruitment. Olmedo et al. [5] demonstrated the presence of free or phagocytosed metal-like particles located in the peri-implant soft tissue, which was suggested, may lead to a corrosive process, as shown by Rodrigues et al. [25] in a retrospective study of implant failure following peri-implantitis. Their subsequent study, which reported on a pyogenic granuloma and peripheral giant cell granuloma associated with dental implants, confirmed the presence and absence of particles, likely titanium, for the former and latter lesions, respectively, leading to the suggestion that PGCG associated with dental implants is simply induced by trauma [5].

In terms of treatment, it is important that any exacerbating factors, such as fractured prostheses and restorations, large or poorly adapted restorations, and dental calculus, should be removed. Other factors should also be considered, such as diastema, which can promote retention of food particles, orthodontic appliances, and dental implants. Following removal of the causal factor, the surgical excision of the lesion should be performed, associated with debridement of the adjacent bone, which is paramount for the prevention of recurrence [9].

Immunohistochemistry for CD68 was performed in order to confirm the origin of the multinucleated giant cells in the lesion. The literature is controversial in terms of the origin of these multinucleated giant cells, with speculation over a macrophage or osteoclast origin. Various studies have shown that multinucleated giant cells in PGCG demonstrate positivity for the immunohistochemical markers MB-1, vimentin, *α*-1-antichymotrypsin, and CD68, suggesting histiocyte/macrophage or osteoclast origin [17, 26]. Galindo-Moreno et al. recently demonstrated that there is no difference between the immunophenotype of the multinucleated giant cells present in PGCG associated with dental implants, when compared to PGCG, central giant cell granuloma, and peri-implant osteolysis [13].

In conclusion, reactive peri-implant lesions should be removed in their entirety in order to prevent recurrence and implant failure. In addition, clinical experience reveals that differences in opinions exist regarding whether the implant should also be removed during excision of the lesion; therefore, it is critical that these lesions are reported so as to arrive at a treatment consensus. Furthermore, it is paramount that histological examination is performed on all peri-implant soft tissue reactions in order to arrive at an appropriate diagnosis, as other neoplastic and nonneoplastic lesions such as pyogenic granuloma, central giant cell granuloma, the brown tumor of hyperparathyroidism, and malignancy must be excluded [27].

## Figures and Tables

**Table 1:** Age, sex, site, initial presentation, time between implant placement and appearance of lesion, treatment, and outcome of cases of PGCG associated with dental implants found in the literature.

Author	Age	Sex	Site	Initial presentation	Type of implant	Time between implant and appearance of the lesion	Recurrence	Treatment
Hirshberg et al. [7]	31	M	Posterior mandible	Peri-implantitis	ND	Unknown	Yes	^*^Curettage/excision (laser)

Hirshberg et al. [7]	44	F	Posterior mandible	Peri-implantitis	ND	6 years	Yes	^*^Curettage/excision and surgical removal of the implant

Hirshberg et al. [7]	69	M	Anterior maxilla	Peri-implantitis	ND	14 months	Yes	^*^Curettage/excision (laser) and surgical removal of the implant

Bischof et al. [8]	56	F	Posterior mandible	Tender rapidly growing mass	Branemark self-tapping Mk II	2 years	No	^*^Excision and curettage

Cloutier et al. [9]	21	M	Posterior mandible	Exophytic mass	Rough-surfaced titanium spray implant	6 years	No	^*^Excision and surgical removal of the implant

Olmedo et al. [5]	64	F	Anterior maxilla	Tumor-like lesion	Branemark-like, 4.1 mm diameter and 11.5 mm length	12 years	No	^*^Excision and curettage

Özden et al. [10]	60	F	Posterior mandible	Exophytic mass	Straumann	6 years	No	^*^Excision and curettage

Hernandez et al. [11]	45	F	Posterior mandible	Bleeding mass	3 self-tapping Branemark implants (3.75 × 7 mm long)	3 years	Yes	^*^Excision and curettage/surgical removal of the implant

Hernandez et al. [11]	36	F	Posterior maxilla	Profuse bleeding on toothbrushing	Branemark self-tapping implant (4 × 13 mm)	2 years	Yes	^*^Excision and curettage/surgical removal of the implant

Hernandez et al. [11]	62	F	Anterior mandible	Exophytic mass	2 self-tapped implants (Branemark system)	3 months	No	^*^Excision and curettage

Peñarrocha-Diago et al. [12]	54	F	Posterior mandible	Exophytic Mass	7 Defcon Avantblast TSA surface implants	2 years	No	^*^Excision and curettage

Galindo-Moreno et al. [13]	74	M	NA	Exophytic mass	NA	6 years	No	^*^Excision and curettage

Present study, 2014	46	M	Posterior mandible	Painless exophytic mass	Neodent Titamax Ex (4 × 11 mm)	10 months	Yes	^*^Excision/excision and curettage

^*^Initial treatment. ND = not disclosed. NA = not available.
